# Modified Multi-Strand Nice Knot Suture Construct Combined with Suture Anchor Fixation Versus Conventional Kirschner Wire Tension Band Fixation for Inferior Patellar Pole Fractures: A Retrospective Comparative Study

**DOI:** 10.3390/jcm15187014

**Published:** 2026-09-10

**Authors:** Junfeng Tang, Chenggang Wang, Xianfa Yuan, Qing Gao, Yuchen Hu, Yusheng Sun, Xiaofeng Liu, Wen Jin, Liangye Sun

**Affiliations:** Department of Orthopedics, Lu’an Hospital of Anhui Medical University, Lu’an 237006, China

**Keywords:** inferior patellar pole fracture, suture anchor, tension band, internal fixation, Nice knot

## Abstract

**Objectives:** To compare the clinical efficacy of the modified multi-strand Nice knot suture construct combined with suture anchor fixation versus conventional Kirschner wire tension band fixation for inferior patellar pole fractures. **Methods:** This retrospective chart review enrolled 108 consecutive patients with inferior patellar pole fractures treated surgically at our institution between January 2019 and January 2024. Patients were allocated to two groups based on the fixation method used: 52 patients underwent the modified suture construct fixation (modified suture fixation group, MSFG), and 56 received conventional Kirschner wire tension band fixation (conventional tension band fixation group, CTBFG). The primary outcomes included anterior knee pain VAS score, knee ROM, and complication rate; secondary outcomes comprised operative time, Bostman, Lysholm and Kujala scores, elective and symptomatic implant removal rates, time to fracture union, Insall–Salvati index, patellar length, and hospitalization costs. **Results:** All surgical procedures were completed uneventfully. Baseline demographic and clinical characteristics were comparable between groups (all *p* > 0.05), with a mean follow-up of 25.5 ± 6.0 months (range, 22–36 months). Operative time did not differ significantly between cohorts (*p* = 0.230). Although the MSFG exhibited statistically lower anterior knee pain VAS scores (0.82 ± 0.6 vs. 1.26 ± 0.8, *p* = 0.003) and slightly greater knee ROM (132.7° ± 9.8° vs. 128.8° ± 8.1°, *p* = 0.027), the magnitude of these differences was below the recognized minimal clinically important difference. At the final follow-up, Bostman, Lysholm and Kujala scores were similar between groups (all *p* > 0.05). Of critical importance, the MSFG had markedly lower rates of implant-related complications and any secondary surgery (both *p* < 0.001). In the CTBFG, 40 patients (71.4%) underwent secondary surgery: 10 for symptomatic hardware irritation and 30 as entirely elective asymptomatic removals. By contrast, no patient in the MSFG required any form of reoperation. No revision surgeries for fixation failure were required in either group. Radiographically, fracture union time and patellar length were comparable (all *p* > 0.05). While the Insall–Salvati index was statistically lower in the MSFG (0.96 ± 0.1 vs. 1.02 ± 0.1, *p* = 0.006), all values remained within the normal physiological range and no functional impairment was observed. Subgroup analysis of comminuted fractures with osteoporosis demonstrated that the MSFG eliminated the implant failure complications seen in the CTBFG (0% vs. 28.6%). **Conclusions:** The modified multi-strand Nice knot suture construct combined with suture anchor fixation provides functional outcomes equivalent to conventional Kirschner wire tension band fixation. However, it offers two distinct advantages: the complete absence of hardware-related complications and the elimination of the need for secondary implant removal. These findings suggest that the modified suture construct may offer particular advantages for patients with comminuted or osteoporotic inferior patellar pole fractures, although further prospective studies are warranted to confirm these observations.

## 1. Introduction

The inferior pole of the patella constitutes the distal cartilaginous-free region, accounting for approximately 25% of the total patellar length. Its primary biomechanical function is to transmit the contractile force of the quadriceps femoris to the tibial tuberosity, thereby facilitating knee extension [1]. Inferior patellar pole fractures are relatively uncommon, representing approximately 11.5% of all patellar fractures, and are classified as extra-articular injuries [2]. The fundamental treatment objectives are to restore extensor mechanism continuity and achieve rigid internal fixation, thereby enabling early functional rehabilitation. For significantly displaced fractures, surgical fixation remains the treatment of choice, with metallic internal fixation being the predominant approach, including Kirschner wire tension band wiring, vertical wire fixation, and plate osteosynthesis [3,4,5]. Although these conventional techniques yield satisfactory clinical outcomes, they frequently necessitate a second surgical procedure for implant removal and are associated with a high incidence of soft tissue irritation and discomfort attributable to prominent metallic hardware.

Numerous studies have demonstrated that suture-based fixation techniques—including transosseous tunnel suturing and suture anchor fixation—achieve favorable clinical outcomes for inferior patellar pole fractures [6,7,8]. Suture fixation eliminates the risk of soft tissue irritation inherent to metallic hardware and obviates the need for secondary implant removal. Furthermore, high-strength braided sutures, such as No. 5 Ethibond and FiberWire, have been shown to possess biomechanical strength comparable to that of metallic wires [9,10,11]. However, in clinical practice, a single fixation construct frequently provides insufficient biomechanical stability for inferior patellar pole fractures complicated by osteoporosis or severe comminution. Consequently, combined fixation strategies are widely recommended for these challenging fracture patterns, including wire augmentation with a patellar concentrator, tension band wiring combined with circumferential cerclage, and suture fixation augmented with suture anchors [12,13,14].

Previous comparative studies have primarily focused on simple transosseous sutures or single suture anchor fixation versus conventional metallic implants. Notably, no study to date has specifically evaluated the clinical efficacy of a modified multi-strand Nice knot construct combined with suture anchor reinforcement—a novel fixation construct that employs a six-strand net-like cradle to rigidly secure the inferior patellar pole fragment and provides enhanced biomechanical stability compared with conventional suture techniques. This retrospective chart review aimed to compare the clinical outcomes, complication profiles, and economic benefits between this modified combined technique and conventional Kirschner wire tension band fixation for inferior patellar pole fractures, thereby providing evidence to inform clinical decision-making.

## 2. Materials and Methods

### 2.1. Study Design and Participants

This retrospective cohort study reviewed consecutive patients with inferior patellar pole fractures treated at our institution between January 2019 and January 2024. Eligible patients were identified through electronic medical records and screened according to predefined inclusion and exclusion criteria. Inclusion criteria were: (1) unilateral acute closed fracture; (2) inferior patellar pole fracture confirmed by radiography or computed tomography without articular cartilage involvement, with significant displacement warranting surgical intervention. Exclusion criteria were: (1) open or pathological fracture; (2) follow-up duration < 12 months; (3) age < 18 years or incomplete clinical data; (4) concomitant fractures or visceral injuries requiring intervention; (5) pre-existing severe degenerative knee disease with functional impairment; and (6) concomitant metabolic disorders or severe medical comorbidities.

**Figure 1:**
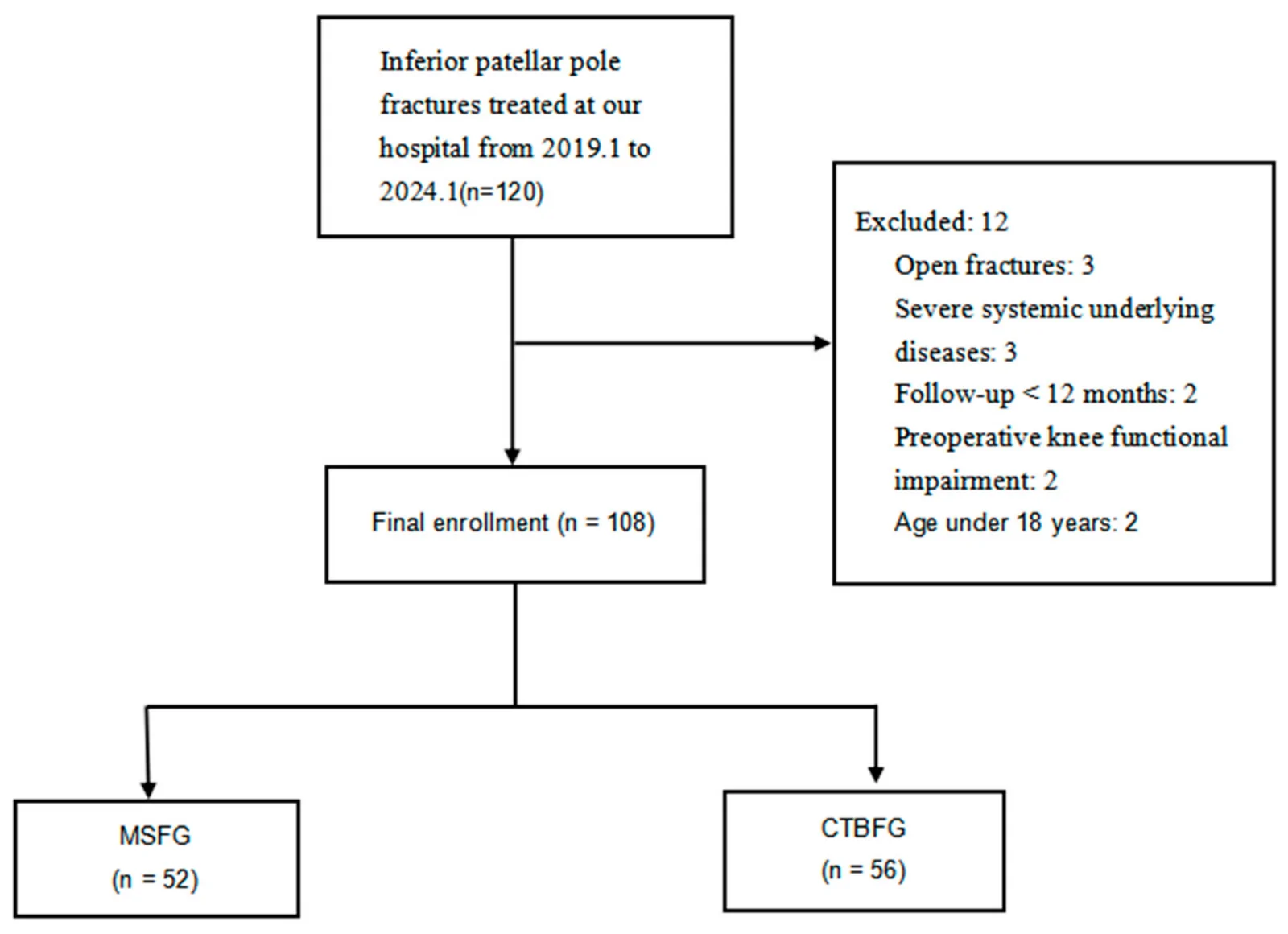
Flowchart of patient enrollment in the study. MSFG: modified suture fixation group. CTBFG: conventional tension band fixation group.

After application of these criteria, 108 patients were included and allocated to two groups: 52 patients underwent modified multi-strand Nice knot suture construct combined with suture anchor fixation (MSFG), and 56 patients underwent conventional Kirschner wire tension band fixation (CTBFG) (Figure 1). Before July 2021, conventional Kirschner wire tension-band fixation (CTBFG) was the standard of care for all eligible patients at our department. After the modified suture construct (MSFG) was introduced in July 2021, surgical decision-making was based on three factors: (1) fracture morphology and bone quality; (2) patient preference and willingness to cover suture anchor costs; and (3) the senior surgeon’s intraoperative assessment of fixation stability. No patient was excluded due to implant unavailability. All surgical procedures were performed by a single experienced senior surgeon. This study was approved by the Institutional Review Board of Lu’an Hospital of Anhui Medical University (NO. 2026LLKS-KY-057).

### 2.2. Surgical Techniques

In the MSFG, a standard anterior midline knee incision was used to expose the fracture site and surrounding soft tissues. The posterior edge of the proximal fracture fragment was divided into four equal segments, with three equidistant points marked. With a 1.5 mm eyelet-tipped Kirschner wire (Wuyang Medical Instrument Co., Ltd., Hefei, China), bone tunnels were drilled from the marked points along the longitudinal patellar axis, angling obliquely toward the anterosuperior patellar edge. A guide suture was passed through each tunnel via the Kirschner wire eyelet, which was then used to shuttle a doubled No. 5 Ethibond (Ethicon, Somerville, NJ, USA) or FiberWire (Arthex Inc., Naples, FL, USA) suture through the tunnel, with the suture loop positioned at the superior patellar pole. (Alternatively, this step can be accomplished by first drilling with a 1.5 mm standard Kirschner wire, followed by suture passage using an Arthrex AR-1291S (Arthex Inc., Naples, FL, USA) nitinol loop suture passer). This configuration yielded six suture strands exiting the distal aspect of the proximal fragment.

**Figure 2:**
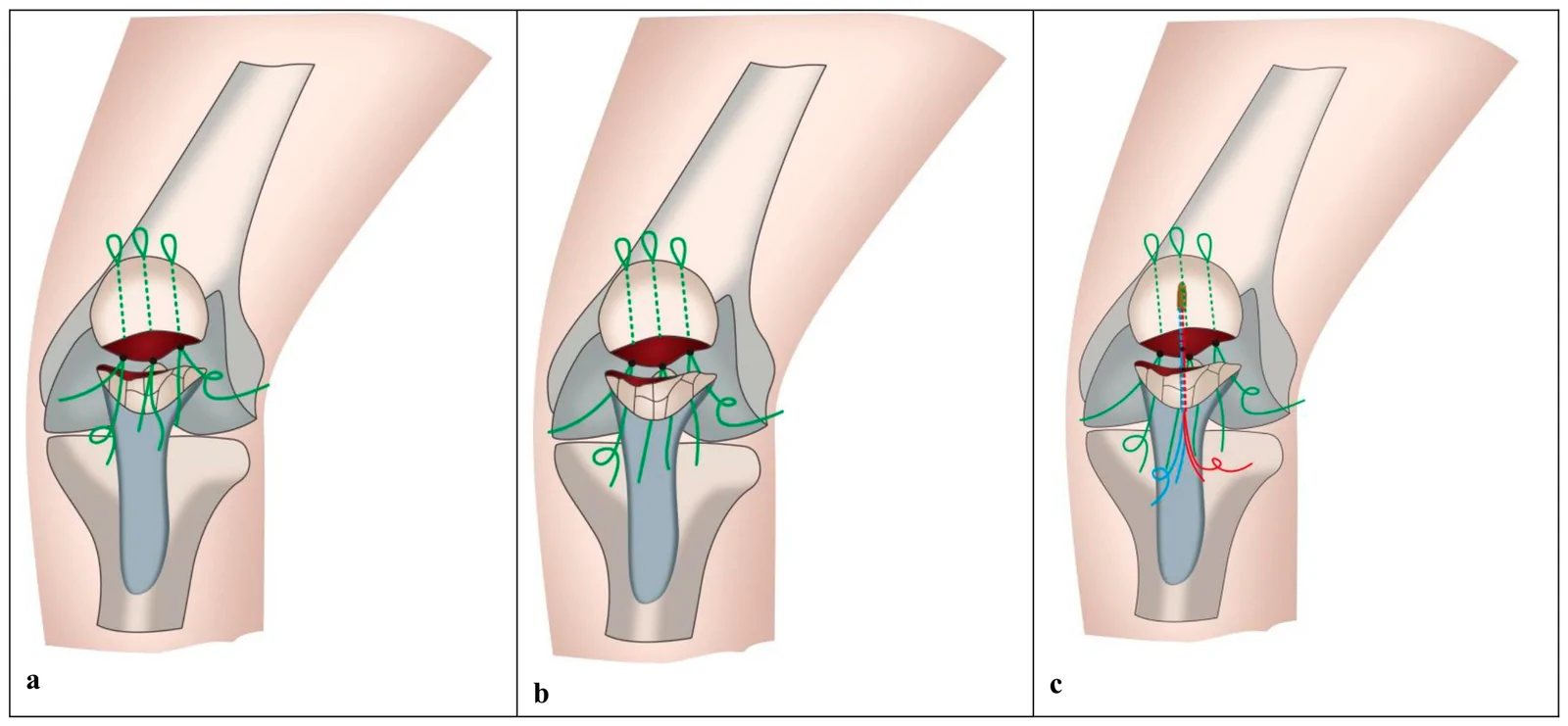

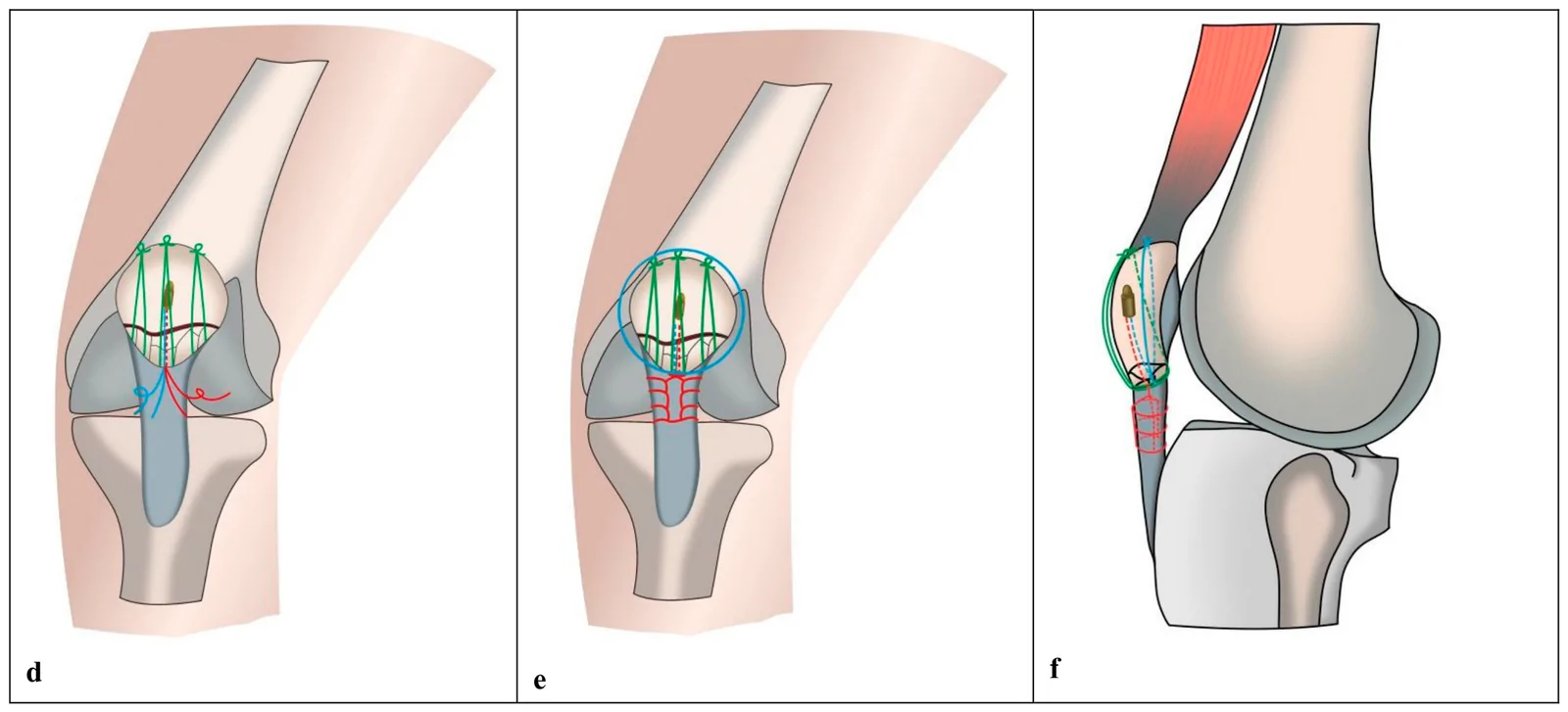
Schematic illustration of the surgical technique for the MSFG. (**a**) The proximal fracture site of the patella is sutured with two parallel sutures, with the fold positioned at the superior edge. (**b**) Six sutures are passed from posterior to anterior in equal intervals through the tendo-patellae junction at the inferior pole of the patella. (**c**) A 5.0 mm suture anchor is passed through the distal fracture site and secured into the proximal fracture site. (**d**) The fracture is reduced, and the sutures are tightened for fixation using a Nice knot. (**e**,**f**) One pair of tail sutures from the anchor is used for a Krackow tension-relieving suture, while another pair is used for circumferential patellar cerclage of the patella.

These six strands were passed equidistantly from posterior to anterior through the bone–tendon junction at the inferior patellar pole. A 5.0 mm suture anchor preloaded with four suture strands (two pairs) was inserted from the inferior pole along the longitudinal patellar axis, slightly off-center, into the proximal patellar fragment. The transosseous sutures were sequentially tightened and secured using modified multi-strand Nice knot constructs with the knee maintained in full extension, achieving anatomic fracture reduction; all knots were positioned at the anterosuperior patellar edge. For additional reinforcement, the remaining anchor suture tails were managed as follows: one pair was woven distally into the mid-distal patellar tendon using the Krackow technique, passed through the tendon, and tied back to the anchor tail; the other pair was used for circumferential patellar cerclage to approximate comminuted peripheral fragments and provide supplementary fixation. To avoid excessive distalization of the patella (patella baja), the Krackow tension-relieving suture in the patellar tendon should be tensioned and tied with the knee held in approximately 30° of flexion. The sequential tightening of the transosseous sutures is performed in full extension. This sequence ensures anatomic reduction without over-tensioning the patellar tendon inferiorly. Satisfactory fracture reduction was confirmed by intraoperative C-arm fluoroscopy, and passive knee range of motion (ROM) testing (flexion ≥ 100°) was performed to verify fixation stability. The prepatellar aponeurosis and parapatellar retinaculum were then closed in layers using 1-0 absorbable sutures (Figure 2).

**Figure 3:**
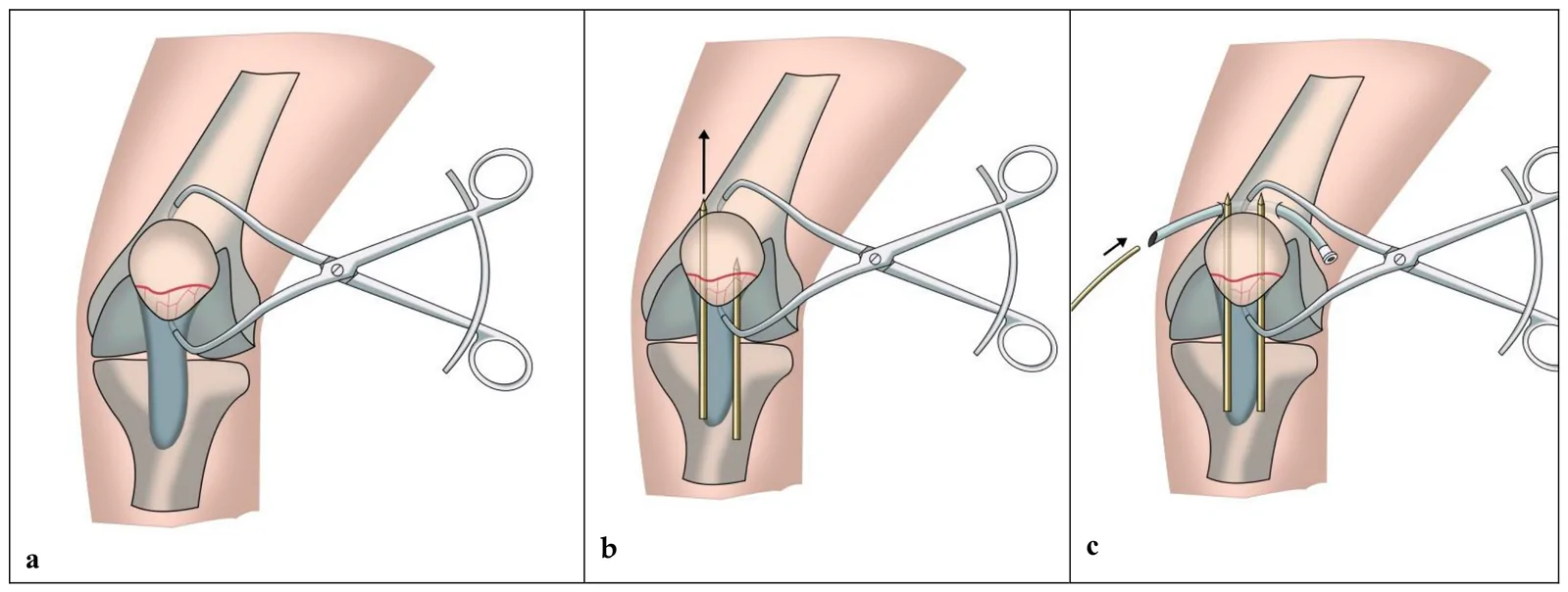

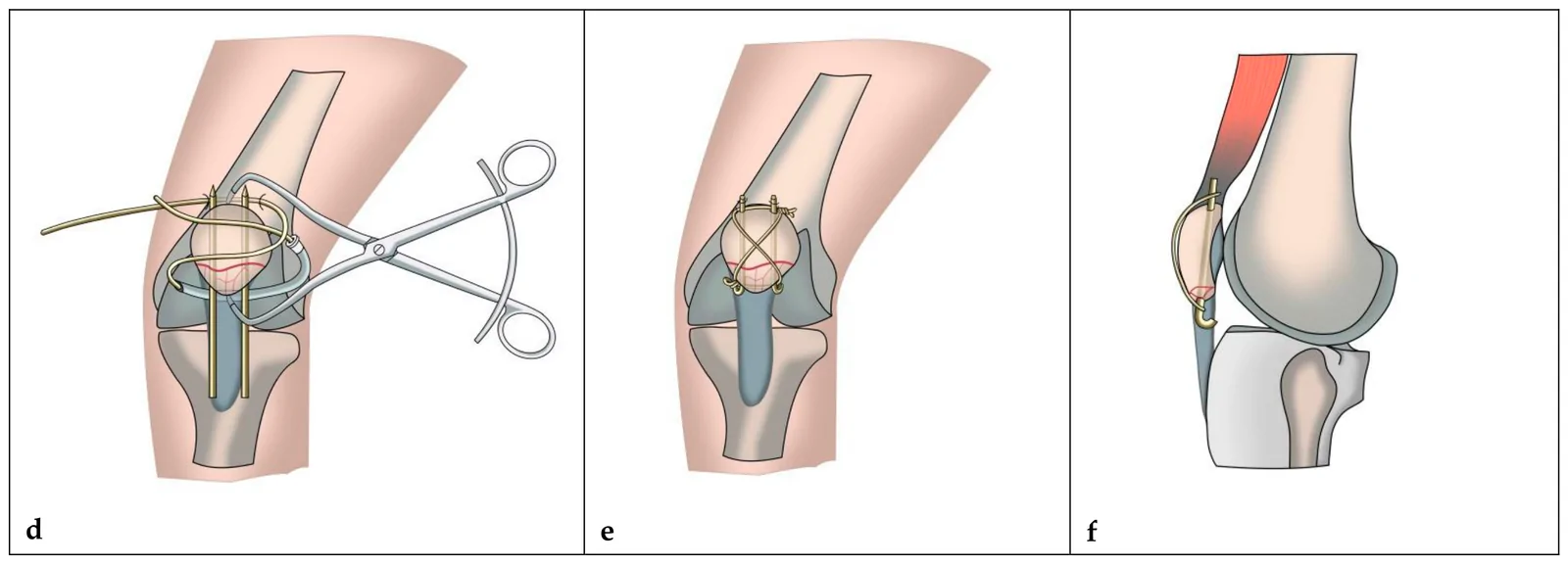
Schematic illustration of the surgical technique for the CTBFG. (**a**) A pointed reduction forceps is applied to maintain reduction in the fracture fragments. (**b**) Two Kirschner wires are drilled into the bone. (**c**,**d**) A figure-of-eight cerclage wiring is performed using stainless steel wire. (**e**,**f**) The wire is tensioned and secured, the Kirschner wires are cut short, and the distal ends of the Kirschner wires are prebent.

In the CTBFG, surgical exposure was identical to that in the MSFG. Two parallel 2.0 mm Kirschner wires were drilled from the distal aspect of the inferior pole proximally, traversing the fracture line and exiting at the superior patellar pole. A stainless steel surgical wire was passed along the trajectory of the two Kirschner wires and configured as a figure-of-eight tension band on the anterior patellar surface. The wire was tightened with the knee held in mild flexion to achieve rigid internal fixation. The protruding ends of the Kirschner wires and the twisted wire knot were cut short, bent, and buried beneath the subcutaneous soft tissues. All subsequent steps were identical to those described for the MSFG (Figure 3).

### 2.3. Postoperative Management

Postoperatively, all patients received intravenous cefazolin (2.0 g) administered 30–60 min before surgical incision. For patients with a history of cephalosporin allergy, intravenous clindamycin (0.6 g) was used as an alternative. Low-molecular-weight heparin combined with intermittent pneumatic compression for thromboprophylaxis. No adjunctive external immobilization was applied to restrict knee ROM. Rehabilitation was initiated on postoperative day 1 under the supervision of a dedicated rehabilitation physician, comprising isometric quadriceps contractions, straight leg raises, and active knee flexion–extension exercises, with partial weight-bearing ambulation using crutches permitted. Progressive active and passive knee ROM exercises were introduced at postoperative week 2, with the goal of achieving full knee flexion by postoperative 6–8 weeks.

### 2.4. Outcome Measures

Data were extracted from electronic medical records, including preoperative baseline characteristics, intraoperative parameters, postoperative functional outcomes, and complications. Baseline characteristics comprised age, gender, body mass index (BMI), osteoporosis, cause of injury, affected side, time from injury to surgery and fracture type. Intraoperative parameters included operative time and implant-related details. The initial hospitalization cost and the secondary surgery hospitalization cost were recorded separately.

Osteoporosis was defined as a bone mineral density T-score ≤ −2.5 at the lumbar spine or femoral neck, measured by dual-energy X-ray absorptiometry within 6 months prior to surgery. In patients without recent DXA data, osteoporosis was diagnosed based on the presence of a fragility fracture combined with age ≥ 65 years and a FRAX score ≥ 10% for major osteoporotic fracture.

Patient-reported outcome measures, collected prospectively as part of the institutional standard protocol, included the anterior knee pain Visual Analog Scale (VAS) score, knee ROM, the Bostman score [15], the Lysholm score [16], and the Kujala score [17]. These validated instruments were administered preoperatively, at 1 and 3 months postoperatively, and annually thereafter. Postoperative follow-up assessments were conducted at 1, 3, 6, and 12 months to evaluate complication rates and profiles.

Radiographic assessment included measurement of the Insall-Salvati index [18] on lateral radiographs to evaluate patellar height and the maximum longitudinal patellar diameter on sagittal images. Fracture union was defined radiographically as complete obliteration of the fracture line with continuous bridging callus across the fracture site. Time to radiographic union was recorded for all patients. Clinical outcomes (ROM, VAS, functional scores) were collected by two independent orthopedic residents not involved in the surgeries, blinded to treatment allocation. Radiographic outcomes (union, Insall-Salvati index, patellar length) were assessed by two senior musculoskeletal radiologists, also blinded to group assignment. Standardized weight-bearing anteroposterior and lateral radiographs were used for all assessments.

The secondary surgeries were categorized into three groups: (1) revision surgery for fixation failure, nonunion, or deep infection; (2) symptomatic implant removal due to hardware-related pain or skin irritation; and (3) elective asymptomatic implant removal solely based on patient request after fracture union. Any secondary surgery was defined as the occurrence of any reoperation, including revision surgery, symptomatic implant removal, or elective asymptomatic implant removal.

### 2.5. Statistical Analysis

All statistical analyses were performed using IBM SPSS Statistics version 23.0 (IBM Corp., Armonk, NY, USA). Continuous variables are expressed as mean ± standard deviation (SD). Normality of data distribution was assessed using the Kolmogorov–Smirnov test. Intergroup comparisons of normally distributed variables were performed using Student’s *t*-test; the Mann–Whitney U test was used for non-normally distributed variables. Categorical variables are presented as frequencies (percentages) and were compared using the chi-squared (χ^2^) test or Fisher’s exact test when expected frequencies were <5 or any cell contained zero counts. All tests were two-tailed, and a *p* value < 0.05 was considered statistically significant. No adjustment was made for multiple comparisons; thus, findings from secondary outcome analyses should be interpreted as exploratory and hypothesis-generating.

## 3. Results

A total of 108 patients were included in the final analysis: 52 patients in the MSFG and 56 patients in the CTBFG. All procedures were completed successfully without intraoperative complications. No significant differences were observed in baseline characteristics between the two groups (Table 1).

All patients were followed for 25.5 ± 6.0 months (range, 22–36 months). No significant difference in operative time was observed between the groups (*p* > 0.05). The MSFG demonstrated significantly lower anterior knee pain VAS scores (0.82 ± 0.6 vs. 1.26 ± 0.8, *p* = 0.003) and significantly greater knee ROM (132.7° ± 9.8° vs. 128.8° ± 8.1°, *p* = 0.027) compared with the CTBFG. Although the MSFG demonstrated statistically significantly lower VAS scores and greater ROM (*p* = 0.003 and *p* = 0.027, respectively), the absolute differences (0.44 points on VAS and 3.9° of flexion) fall below the minimally clinically important differences reported in the literature for these measures. Thus, these differences should be interpreted as statistically significant but not clinically meaningful; both groups achieved an equally excellent functional range.

At final follow-up, no significant between-group differences were found in the Bostman score (29.1 ± 1.3 vs. 28.7 ± 1.1, *p* = 0.092), Lysholm score (95.1 ± 3.7 vs. 94.9 ± 2.8, *p* = 0.753), or Kujala score (94.3 ± 2.1 vs. 93.8 ± 2.9, *p* = 0.304).

The rate of any secondary surgery was significantly lower in the MSFG (0% vs. 71.4%, *p* < 0.001). The initial hospitalization cost was significantly lower in the MSFG than in the CTBFG (13,666.9 ± 761.5 vs. 14,554.5 ± 661.5 CNY, *p* < 0.001). In the CTBFG, the mean secondary surgery hospitalization cost was 3798.2 ± 335.5 CNY, whereas no secondary surgery costs were incurred in the MSFG. The overall complication rate was significantly lower in the MSFG than in the CTBFG (5.8% vs. 32.1%, *p* < 0.001).

Radiographic evaluation revealed no significant between-group differences in time to radiographic union (11.5 ± 1.4 weeks vs. 11.7 ± 1.1 weeks, *p* = 0.068) or maximum longitudinal patellar diameter (47.2 ± 2.8 mm vs. 46.7 ± 3.1 mm, *p* = 0.380). However, the MSFG demonstrated a significantly lower Insall–Salvati index (0.96 ± 0.1 vs. 1.02 ± 0.1, *p* = 0.006) compared with the CTBFG (Table 2).

Complications in the MSFG consisted of two cases of quadriceps atrophy and weakness and one case of asymptomatic patella baja (overall rate 5.8%). In the CTBFG, complications included one case of quadriceps atrophy and weakness, seven cases of implant-related complications, including wire cut-out, loosening, or breakage, none of which required revision surgery, and ten cases of symptomatic hardware irritation necessitating removal. The seven implant-failure patients and the ten symptomatic-removal patients were distinct, yielding a total of 18 patients with complications (32.1%). The seven patients were managed with adjunctive brace immobilization and progressed to union, but all subsequently developed knee stiffness with a reduced final ROM (mean 112°, range 98–122°) compared with the rest of the CTBFG. In the subgroup of patients with comminuted fractures and osteoporosis, no implant-related failures were observed in the MSFG, compared with 28.6% in the CTBFG. However, given the limited subgroup sample size, this finding should be interpreted with caution.

**Figure 4:**
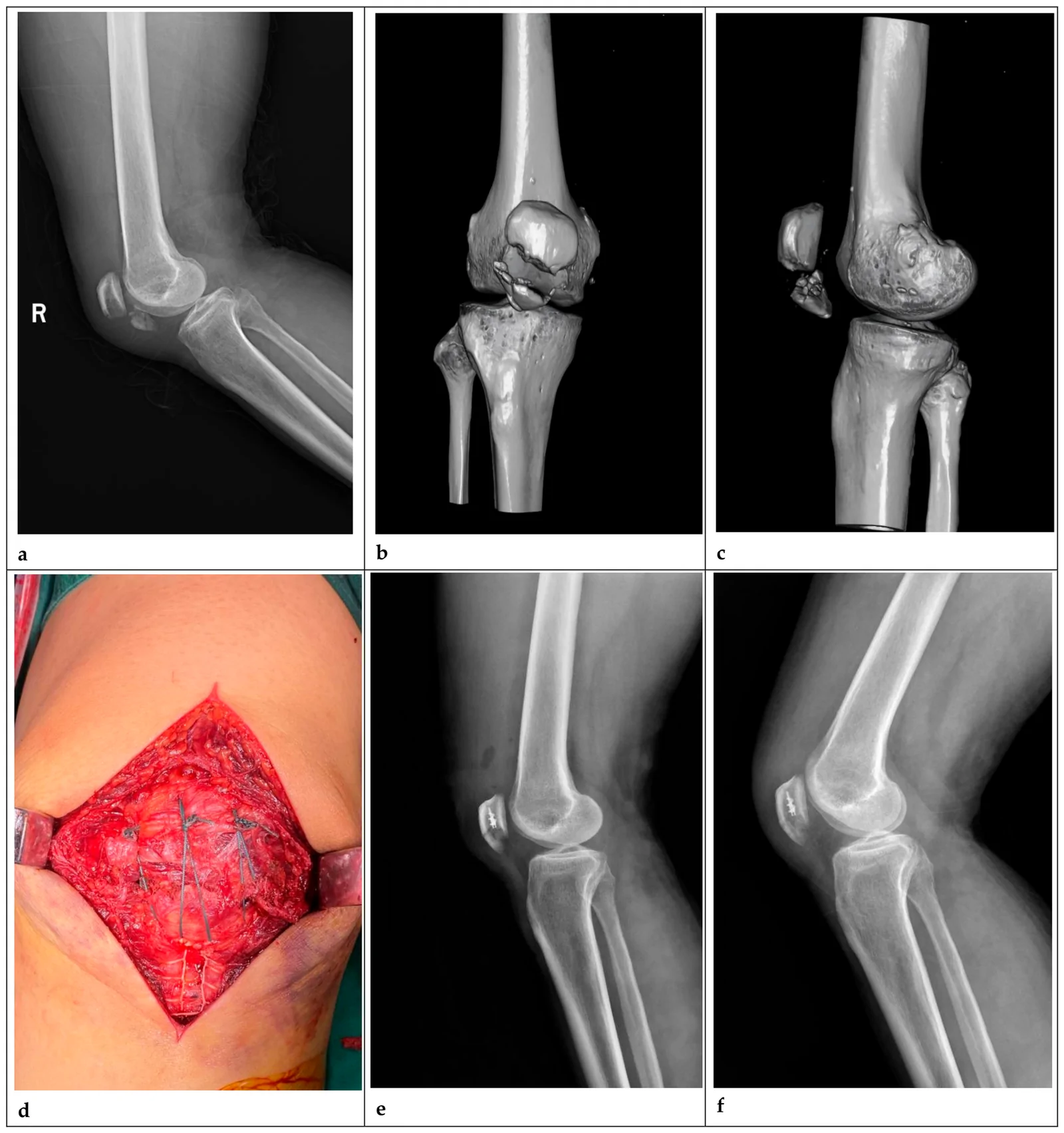
Preoperative and postoperative imaging of a right inferior patellar pole fracture. (**a**–**c**) Preoperative lateral radiographs and three-dimensional computed tomography (CT) images of the right knee show a displaced fracture of the inferior patellar pole. (**d**) Intraoperative view demonstrating reduction and fixation. (**e**) Immediate postoperative lateral radiograph reveals satisfactory fracture reduction and optimal anchor placement. (**f**) At the final follow-up, the lateral radiograph shows solid bone union.

**Figure 5:**
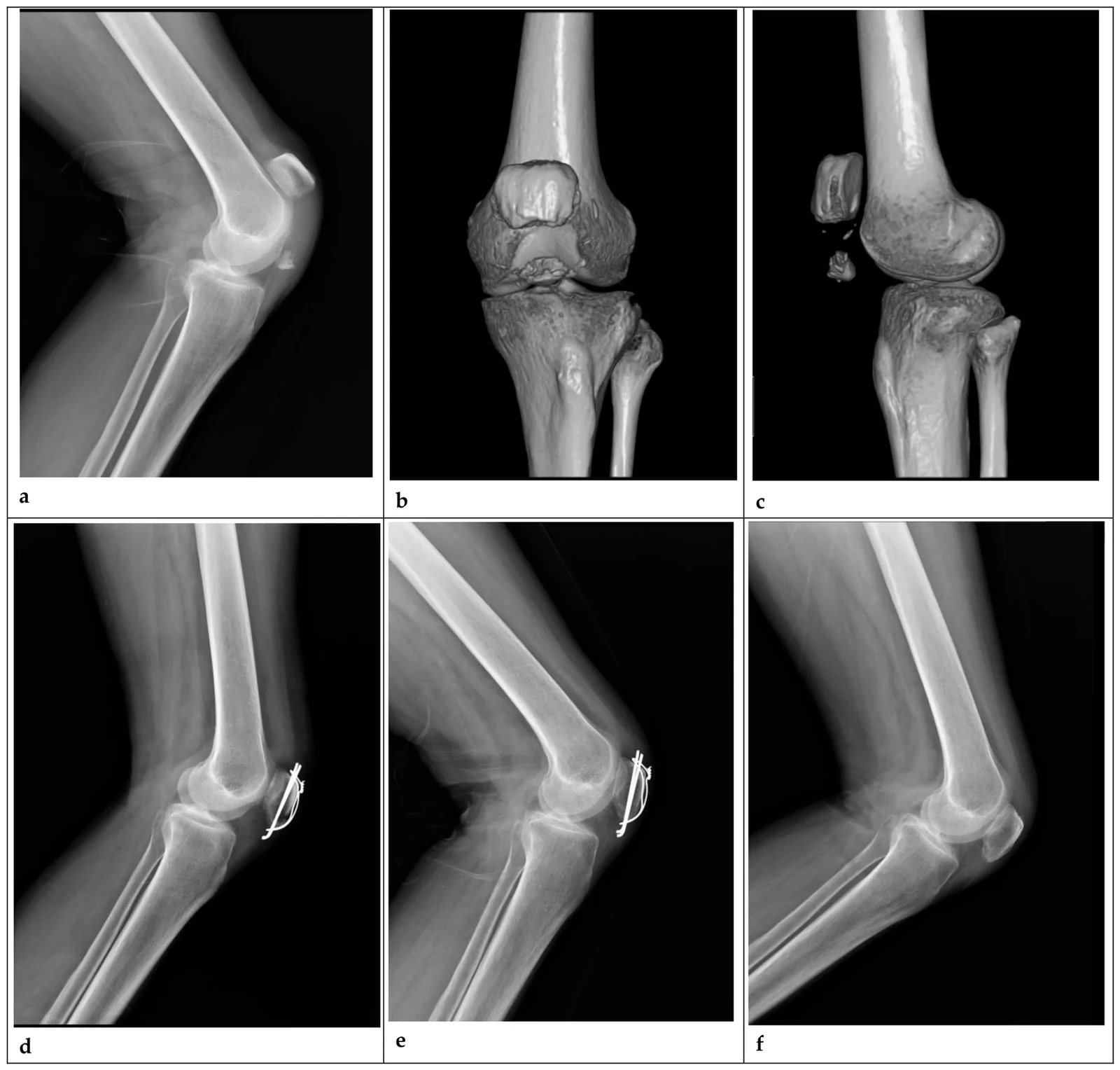
Preoperative and postoperative imaging of a left inferior patellar pole fracture. (**a**–**c**) Preoperative lateral radiographs and three-dimensional computed tomography (CT) images demonstrate a displaced fracture of the left inferior patellar pole. (**d**) The immediate postoperative lateral radiograph shows satisfactory fracture reduction and appropriate positioning of the internal fixation. (**e**) At the 1-year follow-up, the lateral radiograph reveals solid bone union with no evidence of implant loosening. (**f**) After removal of the internal fixation, the lateral radiograph confirms solid bone union.

Radiographic union was achieved in all patients in both groups. No surgical site infections or cases of post-traumatic arthritis were observed in either group. Representative cases are presented in Figure 4 and Figure 5.

## 4. Discussion

The inferior patellar pole serves as the primary insertion site of the patellar tendon and plays a critical biomechanical role in knee extensor mechanism function. Despite the availability of numerous treatment modalities, achieving rigid internal fixation of inferior patellar pole fractures remains technically challenging. Accumulating evidence indicates that single-modality internal fixation is associated with high rates of complications and fixation failure, prompting advocacy for combined fixation strategies to enhance biomechanical stability.

Although conventional metallic fixation techniques, particularly Kirschner wire tension band constructs, remain the clinical mainstay, they are associated with well-documented limitations, including wire cut-out through osteoporotic or comminuted bone fragments, prominent hardware-related soft tissue irritation, and the necessity for secondary implant removal in most patients. These complications not only prolong postoperative rehabilitation but also substantially increase healthcare resource utilization [6,19,20]. Consistent with these reports, all 40 patients who underwent secondary implant removal in our cohort were in the CTBFG, including ten who required removal specifically for symptomatic hardware-related skin irritation. No revision surgeries for implant-related complications or surgical site infection were required in either group.

Furthermore, all seven patients who developed implant-related complications in the CTBFG had comminuted fractures, and six had concomitant osteoporosis. This finding underscores the inherent limitations of conventional metallic fixation in these high-risk populations. In contrast, no implant-related complications were observed in the MSFG. The subgroup analysis focusing on patients with comminuted fractures and osteoporosis starkly illustrates this point. This finding supports the preferential use of the suture construct in osteoporotic bone and comminuted fracture patterns, where the mesh-like, compression-distributing fixation appears biomechanically more lenient.

Recent advances in suture anchor and high-strength suture technologies have expanded the therapeutic armamentarium for inferior patellar pole fractures beyond conventional metallic internal fixation. Numerous studies have demonstrated that suture anchor fixation achieves comparable fracture healing rates and equivalent clinical functional outcomes to metallic fixation [7,21]. Huang et al. [22] reported that both transosseous suturing and suture anchor techniques achieved excellent clinical outcomes in the management of inferior patellar pole fractures. Solunkhe et al. [23] further demonstrated that isolated suture fixation represents a viable alternative to Kirschner wire tension band fixation. Biomechanical studies have confirmed that high-strength braided sutures such as No. 5 Ethibond and FiberWire exhibit tensile strength and clinical fixation performance equivalent to those of stainless steel wires [9,10,11].

Building upon this evidence, we developed a modified vertical interrupted Nice knot construct featuring six suture strands that form a mesh-like cradle to capture the inferior patellar pole, augmented with suture anchor reinforcement. This combined construct provides enhanced biomechanical stability, eliminates the need for postoperative external immobilization, and permits early accelerated rehabilitation under supervised physiotherapy. Consistent with these theoretical advantages, our results demonstrated that although final follow-up functional scores were comparable between groups, the MSFG was associated with significantly lower anterior knee pain VAS scores, greater knee ROM. While we detected statistically significant differences in VAS and ROM favoring the MSFG, the magnitudes were small and below the threshold of clinical relevance. The true clinical superiority of the MSFG lies not in marginally better pain relief or mobility, but in its ability to entirely circumvent the hardware-related adverse events that plagued the CTBFG cohort.

Compared with conventional Kirschner wire tension band fixation, the modified suture–anchor fixation technique described herein offers several distinct advantages. First, the uniform distribution of six suture strands across the inferior patellar pole effectively eliminates stress concentration—a major cause of suture cut-out and fixation failure—while sequential tightening of individual sutures permits precise anatomic reduction in even comminuted fracture fragments. Second, the Nice knot is technically straightforward, features a sliding mechanism that enables progressive tensioning, and achieves secure locking via its double-contact configuration. This knot configuration has been extensively validated for fracture fixation when used with high-strength braided sutures [24,25,26]. The inherent elasticity of these sutures ensures that full tightening of the Nice knot generates uniform compressive forces across the fracture interface; this sustained physiological compression promotes optimal fracture healing. Third, all-suture constructs demonstrate superior biocompatibility compared with metallic implants, completely eliminating the risk of hardware-related soft tissue irritation and obviating the need for secondary implant removal surgery, as corroborated by the absence of implant-related skin irritation in the MSFG.

Two key technical considerations facilitate successful implementation of this technique. Using a 1.5 mm Kirschner wire with an eyelet tip or an Arthrex AR-1291S nitinol wire loop passer simplifies suture passage through bone tunnels, reduces operative time, and enhances accessibility in primary care settings. Additionally, the suture anchor should be inserted through the inferior pole fragment along a trajectory slightly off the central longitudinal patellar axis prior to tightening and tying the vertical transosseous sutures, thereby avoiding anchor impingement or transection of sutures within the bone tunnels, which would compromise fixation stability.

The normal Insall-Salvati index ranges from 0.8 to 1.2, with values <0.8 indicating patella baja and values >1.2 indicating patella alta [18]. Although the MSFG demonstrated a statistically lower Insall-Salvati index, mean values for both groups remained within the normal physiological range, and only one case of mild asymptomatic patella baja was recorded in the MSFG, with no adverse impact on knee function, consistent with the findings of Kim et al. [27]. This slight reduction in the Insall–Salvati index in the MSFG may be attributable to intraoperative tension control during patellar tendon relaxation suturing; careful intraoperative adjustment of suture anchor tension may help maintain physiological patellar height. To mitigate the risk of inadvertent patella baja, we recommend tying the patellar tendon Krackow suture with the knee in 30° of flexion and confirming patellar height fluoroscopically before final knotting. In our series, only one asymptomatic patella baja occurred, and no functional deficits were noted; nevertheless, this technical nuance is crucial during the learning curve.

Analysis of hospitalization costs further reinforced the economic advantage of the MSFG. Although the MSFG already showed a lower initial hospitalization cost, this advantage was further amplified by the complete avoidance of secondary implant removal, which was required in 71.4% of CTBFG patients and generated substantial additional medical and rehabilitation costs. This finding is consistent with Camarda et al. [20], who demonstrated that FiberWire fixation offers superior cost-effectiveness in the treatment of patellar fractures. Therefore, modified suture combined with suture anchor fixation may represent a more economically advantageous alternative for inferior patellar pole fractures.

## 5. Conclusions

For inferior patellar pole fractures, the modified multi-strand Nice knot suture construct with suture anchor fixation achieves functional outcomes equivalent to conventional tension-band wiring. In this retrospective cohort, the suture construct was associated with fewer hardware-related complications and no need for secondary implant removal. These potential benefits appear most pronounced in patients with comminuted fractures and compromised bone quality, but further prospective comparative and biomechanical studies are required to confirm these findings and define its optimal role in clinical practice.

## 6. Research Limitations and Future Directions

This study has several limitations that should be acknowledged. First, as a single-center, non-randomized retrospective cohort study, it carries inherent potential for selection bias. Second, preoperative VAS scores, knee ROM, and functional scores were not systematically compared between groups. Third, we acknowledge that sequential introduction of the new technique introduces potential temporal bias and a learning curve effect, which we have added as an explicit study limitation. Operative time stabilized after the first five MSFG cases, indicating a short learning curve for this experienced surgeon. Finally, the biomechanical performance of this modified fixation construct has not been validated by dedicated in vitro biomechanical testing. Therefore, further large-scale, prospective randomized controlled trials incorporating standardized biomechanical assessments are warranted to confirm and generalize these findings.

## Figures and Tables

**Table 1:** Baseline patient demographics and clinical characteristics. a: χ^2^ test; b: independent samples *t*-test; c: Mann–Whitney U test.

Parameter	MSFG (n = 52)	CTBFG (n = 56)	*p*-Value
Age (years)	57.8 ± 12.2	59.9 ± 14.6	0.417 ^b^
Gender (Male/Female)	30/22	36/20	0.456 ^a^
BMI (kg/m^2^)	22.0 ± 0.8	21.7 ± 0.9	0.069 ^c^
Osteoporosis (present/absent)	18/34	26/30	0.125 ^a^
Cause of injury (low-energy/high-energy)	31/21	38/18	0.428 ^a^
Side (Left/Right)	39/13	40/16	0.661 ^a^
Time from injury to surgery (days)	3.7 ± 0.6	3.9 ± 0.7	0.118 ^c^
Fracture Type (Simple/Comminuted)	9/43	12/44	0.498 ^a^

**Table 2:** Perioperative characteristics and comparison between the two groups. b: independent samples *t*-test; c: Mann–Whitney U test; d: Fisher’s exact test.

Parameter	MSFG (n = 52)	CTBFG (n = 56)	*p*-Value
Follow-up Duration (months)	25.7 ± 6.1	25.2 ± 5.8	0.665 ^b^
Operating time (min)	58.7 ± 10.8	61.0 ± 8.8	0.230 ^b^
VAS score	0.82 ± 0.6	1.26 ± 0.8	0.003 ^c^
ROM	132.7 ± 9.8	128.8 ± 8.1	0.027 ^b^
Bostman score	29.1 ± 1.3	28.7 ± 1.1	0.092 ^c^
Lysholm score	95.1 ± 3.7	94.9 ± 2.8	0.753 ^b^
Kujala score	94.3 ± 2.1	93.8 ± 2.9	0.304 ^b^
Fracture union time (weeks)	11.5 ± 1.4	11.7 ± 1.1	0.068 ^c^
Insall-Salvati index	0.96 ± 0.1	1.02 ± 0.1	0.006 ^c^
Patellar length (mm)	47.2 ± 2.8	46.7 ± 3.1	0.380 ^b^
Secondary surgery (n, %)			
Symptomatic hardware removal	0 (0%)	10 (17.9%)	0.001 ^d^
Elective asymptomatic hardware removal	0 (0%)	30 (53.6%)	<0.001 ^d^
Revision surgery	0 (0%)	0 (0%)	-
Any Secondary surgery	0 (0%)	40 (71.4%)	<0.001 ^d^
Hospitalization Cost (Chinese Yuan, CNY)			
Initial Hospitalization Cost	13,666.9 ± 761.5	14,554.5 ± 661.5	<0.001 ^b^
Secondary surgery Hospitalization Cost	0	3798.2 ± 335.5	-
Complications (n, %)	3 (5.8%)	18 (32.1%)	<0.001 ^d^

## Data Availability

The datasets generated during and/or analyzed during the current study are available from the corresponding author on reasonable request.
